# 4-[(Dimethyl­amino)methyl­idene]-2-(4-nitro­phen­yl)-1,3-oxazol-5(4*H*)-one

**DOI:** 10.1107/S1600536810018635

**Published:** 2010-05-26

**Authors:** Gilberto A. Romeiro, Carlos M. R. Ribeiro, Solange M. S. V. Wardell, James L. Wardell, Seik Weng Ng, Edward R. T. Tiekink

**Affiliations:** a Universidade Federal Fluminense, Departamento de Química Orgãnica, Instituto de Química, Outeiro de São João Baptista, 24020-141 Niterói, RJ, Brazil; b CHEMSOL, 1 Harcourt Road, Aberdeen AB15 5NY, Scotland; c Centro de Desenvolvimento Tecnológico em Saúde (CDTS), Fundação Oswaldo Cruz (FIOCRUZ), Casa Amarela, Campus de Manguinhos, Av. Brasil 4365, 21040-900 Rio de Janeiro, RJ, Brazil; d Department of Chemistry, University of Malaya, 50603 Kuala Lumpur, Malaysia

## Abstract

The title mol­ecule, C_12_H_11_N_3_O_4_, is essentially planar, the r.m.s. deviation for all non-H atoms being 0.068 Å. An intra­molecular C—H⋯N hydrogen bond occurs. The crystal packing is dominated by π–π inter­actions [shortest centroid–centroid distance = 3.6312 (16) Å], which lead to supra­molecular chains that are linked into a three-dimensional network *via* C—H⋯O contacts. The crystal was found to be a non-merohedral twin (twin law −1 0 0/0 −1 0/ 0.784 0 1), the fractional contribution of the minor component being approx­imately 22%.

## Related literature

For the synthesis, synthetic uses and properties of 4-(*N*,*N*-di­methyl­amino­methyl­ene)-2-aryl-2-oxazolin-5-one derivatives, see: Singh & Singh (1994 ▶, 2008 ▶); Takahashi & Izawa (2005 ▶); Singh *et al.* (1994 ▶); Kmetic & Stanovnik (1995 ▶). For the Vilsmeier–Haack reaction, see: Meth-Cohn & Stanforth (1991 ▶). For related structures, see Vasuki *et al.* (2002 ▶); Vijayalakshmi *et al.* (1998 ▶). For the treatment of twinned diffraction data, see: Spek (2009 ▶).
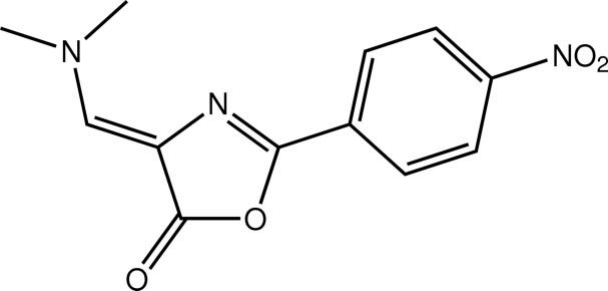


## Experimental

### 

#### Crystal data

C_12_H_11_N_3_O_4_
*M* *_r_* = 261.24
Monoclinic, 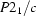

*a* = 9.5313 (2) Å
*b* = 9.5204 (3) Å
*c* = 13.0349 (4) Å
β = 106.661 (2)°
*V* = 1133.15 (6) Å^3^
*Z* = 4
Mo *K*α radiation
μ = 0.12 mm^−1^
*T* = 120 K
0.42 × 0.38 × 0.22 mm

#### Data collection

Nonius KappaCCD area-detector diffractometer
Absorption correction: multi-scan (*SADABS*; Sheldrick, 2007 ▶) *T*_min_ = 0.661, *T*_max_ = 1.000
14210 measured reflections
2581 independent reflections
2030 reflections with *I* > 2σ(*I*)
*R*_int_ = 0.071

#### Refinement

*R*[*F* ^2^ > 2σ(*F* ^2^)] = 0.065
*wR*(*F* ^2^) = 0.220
*S* = 1.19
2581 reflections
176 parameters
H-atom parameters constrained
Δρ_max_ = 0.33 e Å^−3^
Δρ_min_ = −0.30 e Å^−3^

### 

Data collection: *COLLECT* (Hooft, 1998 ▶); cell refinement: *DENZO* (Otwinowski & Minor, 1997 ▶) and *COLLECT*; data reduction: *DENZO* and *COLLECT*; program(s) used to solve structure: *SHELXS97* (Sheldrick, 2008 ▶); program(s) used to refine structure: *SHELXL97* (Sheldrick, 2008 ▶); molecular graphics: *ORTEP-3* (Farrugia, 1997 ▶) and *DIAMOND* (Brandenburg, 2006 ▶); software used to prepare material for publication: *publCIF* (Westrip, 2010 ▶).

## Supplementary Material

Crystal structure: contains datablocks global, I. DOI: 10.1107/S1600536810018635/ez2209sup1.cif

Structure factors: contains datablocks I. DOI: 10.1107/S1600536810018635/ez2209Isup2.hkl

Additional supplementary materials:  crystallographic information; 3D view; checkCIF report

## Figures and Tables

**Table 1:** Hydrogen-bond geometry (Å, °)

*D*—H⋯*A*	*D*—H	H⋯*A*	*D*⋯*A*	*D*—H⋯*A*
C5—H5c⋯N1	0.98	2.28	3.074 (5)	137
C5—H5a⋯O2^i^	0.98	2.53	3.504 (4)	177
C5—H5c⋯O4^ii^	0.98	2.57	3.259 (5)	127
C9—H9⋯O1^iii^	0.95	2.56	3.304 (4)	135
C11—H11⋯O2^iv^	0.95	2.45	3.144 (4)	130

Symmetry codes: (i) 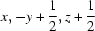; (ii) 
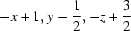
; (iii) 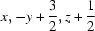; (iv) 
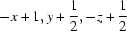
.

**Fig. 1.**
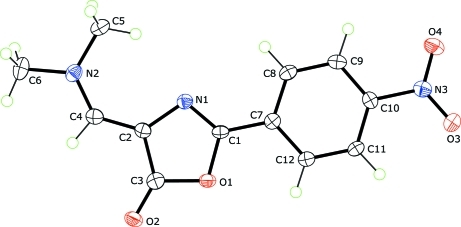
The molecular structure of (I) showing the atom-labelling scheme and displacement ellipsoids at the 50% probability level.

**Fig. 2.**
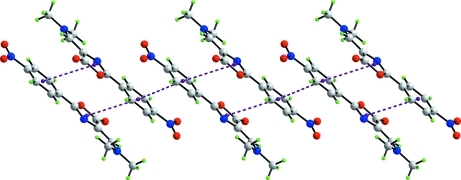
A view of the supramolecular chain aligned along the b axis in (I) sustained by π–π intercations (purple dashed lines). Colour code: O, red; N, blue; C, grey; and H, green.

**Fig. 3.**
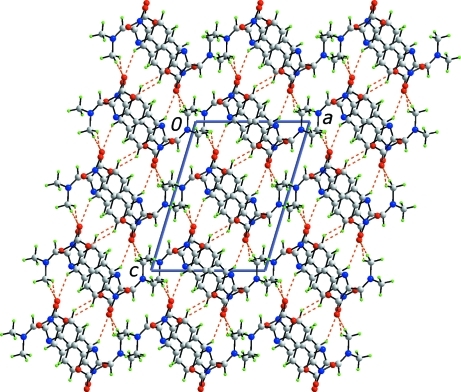
View of the connections between chains in (I) with the C–H···O interactions shown as orange dashed lines. Colour code: O, red; N, blue; C, grey; and H, green.

## supplementary crystallographic information

### Comment

The preparations of 4-(*N*,*N*-dimethylaminomethylene)-2-aryl-2-oxazolin-5-one derivatives have been reported using the Vilsmeier-Haack reactions (Meth-Cohn & Stanforth, 1991) of acylaminoacetanilides with POCl_3_ and DMF (Singh & Singh, 1994; Takahashi & Izawa, 2005; Singh *et al.*, 1994; Kmetic & Stanovnik, 1995). The compounds have been used as precursors of 4-hydroxymethylene-2-aryl-2-oxazolin-5-one, which have been tested for anti-bacterial activities (Singh & Singh, 2008). The crystal structures of 4-(*N*,*N*-dimethylaminomethylene)-2-phenyl-2-oxazolin-5-one (Vasuki *et al.*, 2002) and 4-(*N*,*N*-dimethylaminomethylene)-2-(2-nitrophenyl)-2-oxazolin-5-one (Vijayalakshmi *et al.*, 1998) have been reported. We now report the crystal structure of 4-(*N*,*N*-dimethylaminomethylene)-2-(4-nitrophenyl)-2-oxazolin-5-one, (I).

The molecule of (I), Fig. 1, is essentially planar with the maximum deviations from the least-squares plane through all non-hydrogen atoms being 0.157 (4) Å for atom C5 and -0.158 (3) for atom O4; the r.m.s. = 0.068 Å. The sequence of C1–N1, N1–C2, C2–C4, and C4–N2 bond distances of 1.289 (4), 1.398 (4), 1.382 (5), and 1.317 (4) Å, respectively, indicate substantial delocalisation of π-electron density over these atoms. The geometric parameters in (I) match closely those found in the parent compound, namely 4-(*N*,*N*-dimethylaminomethylene)-2-phenyl-2-oxazolin-5-one (Vasuki *et al.*, 2002) and in the 2-nitro derivative (Vijayalakshmi *et al.*, 1998).

The crystal packing is dominated by C–H···O and π–π interactions; the N1 atom of the oxazolin-5-one is involved in an intramolecular C–H···N contact that shields this atom from forming intermolecular interactions, Table 1. Columns of molecules orientated along the *b* axis are stabilised by π–π contacts with the shortest of these occurring between centrosymmetrically related benzene rings [ring centroid(C7–C12)···ring centroid(C7–C12)^i^ = 3.6312 (16) Å for *i*: 1-*x*, 1-*y*, 2-*z*]. The benzene rings also form π–π interactions with the oxazolin-5-one rings [ring centroid(C7–C12)···ring centroid(O1,N1,C1–C3)^ii^ = 3.7645 (17) Å for *ii*: 1-*x*, -*y*, 2-*z*] to form a supramolecular chain, Fig. 2. The chains are connected by a series of C–H···O contacts, Table 1, to form a 3-D network, Fig. 3.

### Experimental

The title compound was prepared as per published procedures (Singh & Singh, 1994; Singh *et al.*, 1994). Physical properties were in agreement with published data. The crystal used in the structure determination was grown from EtOH solution.

### Refinement

The C-bound H atoms were geometrically placed (C–H = 0.95–0.98 Å) and refined as riding with *U*_iso_(H) = 1.2–1.5*U*_eq_(C). For the treatment of twinned diffraction data, see: Spek (2009).

### Crystal data

**Table:**

C_12_H_11_N_3_O_4_	*F*(000) = 544
*M**_r_* = 261.24	*D*_x_ = 1.531 Mg m^−^^3^
Monoclinic, *P*2_1_/*c*	Mo *K*α radiation, λ = 0.71073 Å
Hall symbol: -P 2ybc	Cell parameters from 2714 reflections
*a* = 9.5313 (2) Å	θ = 2.9–27.5°
*b* = 9.5204 (3) Å	µ = 0.12 mm^−^^1^
*c* = 13.0349 (4) Å	*T* = 120 K
β = 106.661 (2)°	Block, red
*V* = 1133.15 (6) Å^3^	0.42 × 0.38 × 0.22 mm
*Z* = 4	

### Data collection

**Table:**

Nonius KappaCCD area-detector diffractometer	2581 independent reflections
Radiation source: Enraf Nonius FR591 rotating anode	2030 reflections with *I* > 2σ(*I*)
10 cm confocal mirrors	*R*_int_ = 0.071
Detector resolution: 9.091 pixels mm^-1^	θ_max_ = 27.4°, θ_min_ = 3.1°
φ and ω scans	*h* = −12→12
Absorption correction: multi-scan (*SADABS*; Sheldrick, 2007)	*k* = −12→11
*T*_min_ = 0.661, *T*_max_ = 1.000	*l* = −16→16
14210 measured reflections	

### Refinement

**Table:**

Refinement on *F*^2^	Secondary atom site location: difference Fourier map
Least-squares matrix: full	Hydrogen site location: inferred from neighbouring sites
*R*[*F*^2^ > 2σ(*F*^2^)] = 0.065	H-atom parameters constrained
*wR*(*F*^2^) = 0.220	*w* = 1/[σ^2^(*F*_o_^2^) + (0.0936*P*)^2^ + 1.6594*P*] where *P* = (*F*_o_^2^ + 2*F*_c_^2^)/3
*S* = 1.19	(Δ/σ)_max_ = 0.001
2581 reflections	Δρ_max_ = 0.33 e Å^−^^3^
176 parameters	Δρ_min_ = −0.30 e Å^−^^3^
0 restraints	Extinction correction: *SHELXL97* (Sheldrick, 2008), Fc^*^=kFc[1+0.001xFc^2^λ^3^/sin(2θ)]^-1/4^
Primary atom site location: structure-invariant direct methods	Extinction coefficient: 0.018 (5)

### Special details

**Table:**

Geometry. All esds (except the esd in the dihedral angle between two l.s. planes) are estimated using the full covariance matrix. The cell esds are taken into account individually in the estimation of esds in distances, angles and torsion angles; correlations between esds in cell parameters are only used when they are defined by crystal symmetry. An approximate (isotropic) treatment of cell esds is used for estimating esds involving l.s. planes.
Refinement. Refinement of *F*^2^ against ALL reflections. The weighted *R*-factor wR and goodness of fit *S* are based on *F*^2^, conventional *R*-factors *R* are based on *F*, with *F* set to zero for negative *F*^2^. The threshold expression of *F*^2^ > 2σ(*F*^2^) is used only for calculating *R*-factors(gt) etc. and is not relevant to the choice of reflections for refinement. *R*-factors based on *F*^2^ are statistically about twice as large as those based on *F*, and *R*- factors based on ALL data will be even larger.

### Fractional atomic coordinates and isotropic or equivalent isotropic displacement parameters (Å^2^)

**Table:**

	*x*	*y*	*z*	*U*_iso_*/*U*_eq_	
O1	0.3806 (2)	0.5986 (2)	0.33548 (17)	0.0197 (5)	
O2	0.2556 (3)	0.4481 (2)	0.20576 (17)	0.0239 (6)	
O3	0.8419 (3)	1.1001 (3)	0.6327 (2)	0.0324 (6)	
O4	0.7443 (3)	1.0746 (3)	0.76142 (19)	0.0307 (6)	
N1	0.2875 (3)	0.5457 (3)	0.4711 (2)	0.0180 (6)	
N2	0.0528 (3)	0.3059 (3)	0.4441 (2)	0.0203 (6)	
N3	0.7556 (3)	1.0430 (3)	0.6733 (2)	0.0209 (6)	
C1	0.3786 (3)	0.6220 (3)	0.4393 (2)	0.0166 (6)	
C2	0.2186 (3)	0.4617 (3)	0.3831 (2)	0.0179 (6)	
C3	0.2761 (3)	0.4921 (3)	0.2958 (2)	0.0195 (7)	
C4	0.1130 (3)	0.3590 (3)	0.3735 (2)	0.0189 (7)	
H4	0.0778	0.3199	0.3038	0.023*	
C5	0.0939 (4)	0.3462 (4)	0.5569 (3)	0.0237 (7)	
H5A	0.1378	0.2655	0.6012	0.036*	
H5B	0.0066	0.3768	0.5761	0.036*	
H5C	0.1649	0.4233	0.5691	0.036*	
C6	−0.0548 (4)	0.1947 (4)	0.4138 (3)	0.0284 (8)	
H6A	−0.0780	0.1778	0.3366	0.043*	
H6B	−0.1440	0.2223	0.4317	0.043*	
H6C	−0.0152	0.1086	0.4526	0.043*	
C7	0.4765 (3)	0.7290 (3)	0.4994 (2)	0.0168 (6)	
C8	0.4797 (3)	0.7571 (3)	0.6051 (2)	0.0184 (6)	
H8	0.4184	0.7056	0.6375	0.022*	
C9	0.5715 (3)	0.8590 (3)	0.6624 (2)	0.0185 (6)	
H9	0.5735	0.8794	0.7342	0.022*	
C10	0.6608 (3)	0.9314 (3)	0.6135 (2)	0.0178 (6)	
C11	0.6608 (3)	0.9058 (3)	0.5089 (2)	0.0173 (6)	
H11	0.7231	0.9571	0.4772	0.021*	
C12	0.5676 (3)	0.8035 (3)	0.4519 (2)	0.0175 (6)	
H12	0.5655	0.7838	0.3800	0.021*	

### Atomic displacement parameters (Å^2^)

**Table:**

	*U*^11^	*U*^22^	*U*^33^	*U*^12^	*U*^13^	*U*^23^
O1	0.0239 (12)	0.0203 (11)	0.0177 (11)	−0.0022 (9)	0.0105 (9)	−0.0010 (8)
O2	0.0307 (13)	0.0242 (12)	0.0187 (11)	−0.0017 (10)	0.0100 (10)	−0.0029 (9)
O3	0.0323 (14)	0.0382 (15)	0.0291 (13)	−0.0147 (12)	0.0127 (11)	−0.0044 (11)
O4	0.0397 (15)	0.0328 (14)	0.0219 (12)	−0.0061 (12)	0.0125 (11)	−0.0075 (10)
N1	0.0195 (13)	0.0171 (12)	0.0183 (13)	0.0003 (10)	0.0067 (10)	0.0004 (10)
N2	0.0209 (13)	0.0163 (13)	0.0248 (13)	0.0015 (11)	0.0082 (11)	0.0025 (10)
N3	0.0219 (13)	0.0210 (13)	0.0195 (13)	0.0017 (12)	0.0056 (11)	0.0024 (11)
C1	0.0193 (14)	0.0179 (14)	0.0143 (13)	0.0046 (12)	0.0073 (11)	0.0029 (11)
C2	0.0198 (15)	0.0172 (14)	0.0173 (14)	0.0032 (12)	0.0065 (12)	0.0009 (11)
C3	0.0218 (15)	0.0165 (14)	0.0207 (15)	0.0022 (12)	0.0068 (12)	0.0030 (12)
C4	0.0222 (16)	0.0156 (14)	0.0198 (15)	0.0042 (12)	0.0076 (12)	0.0024 (11)
C5	0.0270 (17)	0.0235 (16)	0.0246 (16)	0.0024 (14)	0.0136 (14)	0.0033 (13)
C6	0.0247 (17)	0.0210 (16)	0.039 (2)	−0.0050 (14)	0.0077 (15)	0.0059 (14)
C7	0.0182 (15)	0.0145 (14)	0.0185 (14)	0.0035 (12)	0.0062 (12)	0.0022 (11)
C8	0.0201 (15)	0.0188 (15)	0.0184 (14)	0.0018 (12)	0.0089 (12)	0.0040 (12)
C9	0.0215 (15)	0.0193 (15)	0.0158 (13)	0.0052 (13)	0.0070 (12)	0.0030 (12)
C10	0.0174 (14)	0.0152 (14)	0.0198 (15)	0.0025 (12)	0.0036 (12)	−0.0005 (11)
C11	0.0180 (14)	0.0175 (14)	0.0178 (14)	0.0023 (12)	0.0073 (11)	0.0035 (11)
C12	0.0193 (14)	0.0184 (14)	0.0169 (14)	0.0029 (12)	0.0086 (12)	0.0014 (11)

### Geometric parameters (Å, °)

**Table:**

O1—C1	1.377 (3)	C5—H5B	0.9800
O1—C3	1.411 (4)	C5—H5C	0.9800
O2—C3	1.209 (4)	C6—H6A	0.9800
O3—N3	1.226 (4)	C6—H6B	0.9800
O4—N3	1.222 (4)	C6—H6C	0.9800
N1—C1	1.289 (4)	C7—C8	1.394 (4)
N1—C2	1.398 (4)	C7—C12	1.396 (4)
N2—C4	1.317 (4)	C8—C9	1.375 (4)
N2—C6	1.448 (4)	C8—H8	0.9500
N2—C5	1.460 (4)	C9—C10	1.385 (4)
N3—C10	1.466 (4)	C9—H9	0.9500
C1—C7	1.450 (4)	C10—C11	1.385 (4)
C2—C4	1.382 (5)	C11—C12	1.383 (4)
C2—C3	1.428 (4)	C11—H11	0.9500
C4—H4	0.9500	C12—H12	0.9500
C5—H5A	0.9800		
			
C1—O1—C3	105.6 (2)	H5B—C5—H5C	109.5
C1—N1—C2	105.0 (2)	N2—C6—H6A	109.5
C4—N2—C6	120.5 (3)	N2—C6—H6B	109.5
C4—N2—C5	123.9 (3)	H6A—C6—H6B	109.5
C6—N2—C5	115.5 (3)	N2—C6—H6C	109.5
O4—N3—O3	123.2 (3)	H6A—C6—H6C	109.5
O4—N3—C10	118.1 (3)	H6B—C6—H6C	109.5
O3—N3—C10	118.7 (3)	C8—C7—C12	120.0 (3)
N1—C1—O1	115.2 (3)	C8—C7—C1	119.8 (3)
N1—C1—C7	127.6 (3)	C12—C7—C1	120.2 (3)
O1—C1—C7	117.2 (3)	C9—C8—C7	120.2 (3)
C4—C2—N1	129.6 (3)	C9—C8—H8	119.9
C4—C2—C3	120.5 (3)	C7—C8—H8	119.9
N1—C2—C3	109.9 (3)	C8—C9—C10	118.7 (3)
O2—C3—O1	120.4 (3)	C8—C9—H9	120.7
O2—C3—C2	135.4 (3)	C10—C9—H9	120.7
O1—C3—C2	104.3 (2)	C11—C10—C9	122.7 (3)
N2—C4—C2	131.3 (3)	C11—C10—N3	118.5 (3)
N2—C4—H4	114.4	C9—C10—N3	118.8 (3)
C2—C4—H4	114.4	C12—C11—C10	118.1 (3)
N2—C5—H5A	109.5	C12—C11—H11	120.9
N2—C5—H5B	109.5	C10—C11—H11	120.9
H5A—C5—H5B	109.5	C11—C12—C7	120.4 (3)
N2—C5—H5C	109.5	C11—C12—H12	119.8
H5A—C5—H5C	109.5	C7—C12—H12	119.8
			
C2—N1—C1—O1	−0.3 (3)	O1—C1—C7—C8	−179.8 (3)
C2—N1—C1—C7	179.1 (3)	N1—C1—C7—C12	−179.3 (3)
C3—O1—C1—N1	−0.1 (3)	O1—C1—C7—C12	0.1 (4)
C3—O1—C1—C7	−179.5 (3)	C12—C7—C8—C9	0.6 (5)
C1—N1—C2—C4	178.8 (3)	C1—C7—C8—C9	−179.5 (3)
C1—N1—C2—C3	0.5 (3)	C7—C8—C9—C10	−0.7 (5)
C1—O1—C3—O2	−178.9 (3)	C8—C9—C10—C11	0.4 (5)
C1—O1—C3—C2	0.4 (3)	C8—C9—C10—N3	178.2 (3)
C4—C2—C3—O2	0.1 (6)	O4—N3—C10—C11	172.7 (3)
N1—C2—C3—O2	178.6 (4)	O3—N3—C10—C11	−7.1 (4)
C4—C2—C3—O1	−179.1 (3)	O4—N3—C10—C9	−5.1 (4)
N1—C2—C3—O1	−0.6 (3)	O3—N3—C10—C9	175.0 (3)
C6—N2—C4—C2	−178.4 (3)	C9—C10—C11—C12	−0.1 (5)
C5—N2—C4—C2	−2.4 (5)	N3—C10—C11—C12	−177.9 (3)
N1—C2—C4—N2	−3.9 (6)	C10—C11—C12—C7	0.1 (4)
C3—C2—C4—N2	174.2 (3)	C8—C7—C12—C11	−0.3 (4)
N1—C1—C7—C8	0.9 (5)	C1—C7—C12—C11	179.8 (3)

### Hydrogen-bond geometry (Å, °)

**Table:**

*D*—H···*A*	*D*—H	H···*A*	*D*···*A*	*D*—H···*A*
C5—H5c···N1	0.98	2.28	3.074 (5)	137
C5—H5a···O2^i^	0.98	2.53	3.504 (4)	177
C5—H5c···O4^ii^	0.98	2.57	3.259 (5)	127
C9—H9···O1^iii^	0.95	2.56	3.304 (4)	135
C11—H11···O2^iv^	0.95	2.45	3.144 (4)	130

Symmetry codes:  (i) *x*, −*y*+1/2, *z*+1/2; (ii) −*x*+1, *y*−1/2, −*z*+3/2; (iii) *x*, −*y*+3/2, *z*+1/2; (iv) −*x*+1, *y*+1/2, −*z*+1/2.
